# Supplementary material for: Compensated Advanced Chronic Liver Disease and Steatosis in Patients with Type 2 Diabetes as Assessed through Shear Wave Measurements and Attenuation Measurements
Source: Biomedicines. 2024 Jan 30;12(2):323. doi: 10.3390/biomedicines12020323 (PMC10886655; doi:10.3390/biomedicines12020323)
Supplement: Supplementary file 1 [file biomedicines-12-00323-s001.zip › Supplementary Table S1.pdf]

Supplementary Table S1. Univariate and multivariate logistic regression model assessing independent predictors associated with the SWM>8.3 kPa (as the indicator of suspicion of cACLD). Significant p-values are bold. **Abbreviations** ALT: alanine aminotransferase; AST: aspartate aminotransferase; ALP: Alkaline phosphatase; APRI: AST to Platelet Ratio Index; ATT: Attenuation coefficient measurement**;** CAP: continuous attenuation parameter; cACLD: compensated advanced chronic liver disease, CRP: C-reactive protein; dB/cm/MHz: decibel per centimeter per megahertz; dB/m: decibels per meter; FIB-4: Fibrosis-4; GGT: gamma glutamyl transferase; g/L: grams per liter; HbA1c: glycated haemoglobin; HDL: high-density lipoprotein; IQR: interquartile range; kPa: kilopascal; LDL: low-density lipoprotein; M: median; µmol/L: micromoles per liter; mmol/L: millimoles per liter; N: number; MASLD: Metabolic dysfunction-associated steatotic liver disease; PT: prothrombin time; SCD: skin to capsule distance; SWM: shear wave measurement; U/L: units per liter; VCTE: vibration-controlled transient elastography; Vs: shear wave speed.

**Dependent variable: SWM>8,3 kPa**	**Odds ratio (univariate)**	**95% CI**	**Odds ratio (multivariate)**	**95% CI**	**P value for multivariate analysis**
**Independent variables:**					
**Age, years**	0.993	0.961-1.025			
**Male sex**	1.418	0.691-2.911			
**BMI, kg/m ^{2}**	0.978	0.912-1.048			
**Obesity (BMI>30 kg/m2)**	1.04	0.503-2.15			
**Arterial hypertension**	0.578	0.26-1.285			
**Hyperlipidemia**	0.839	0.374-1.877			
**Smoking**	**2.868**	**1.347-6.103**	2.242	0.556-10.561	0.24
**Hematocrit**	81.433	0.057-116817.19			
**Red cell count, G/L**	0.603	0.257-1.417			
**Platelets, G/L**	**0.978**	**0.969-0.987**	**0.973**	**0.948-0.998**	**0.04**
**PT (%)**	0.989	0.964-1.015			
**Glucose, mmol/L**	1.001	0.88-1.14			
**HbA1c (%)**	0.829	0.606-1.133			
**Creatinine, µmol/L**	0.988	0.971-1.004			

**AST, U/L**	**1.071**	**1.043-1.100**	**1.195**	**1.026-1.392**	**0.02**
**ALT, U/L**	**1.015**	**1.003-1.027**	0.97	0.934-1.008	0.12
**GGT, U/L**	1.002	0.999-1.005			
**ALP, U/L**	1.002	0.994-1.01			
**Total cholesterol, mmol/L**	1.161	0.884-1.525			
**Triglycerides, mmol/L**	0.634	0.398-1.009			
**HDL, mmol/L**	3.009	0.987-9.18			
**LDL, mmol/L**	1.216	0.839-1.764			
**Albumins, g/L**	1.055	0.974-1.143			
**CRP, mg/L, mmol/L**	1.017	0.949-1.09			
**NAFLD fibrosis score, points**	0.237	0.014-4.033			
**FIB-4, points**	**5.778**	**3.002-11.124**	0.579	0.112-2.987	0.51
**APRI, points**	**849.463**	**84.784-8510.933**	0.002	0-289.514	0.31
**Fibroscan XL probe**	1.056	0.495-2.255			
**LSM by VCTE, kPa**	**1.521**	**1.307-1.77**	**1.376**	**1.16-1.632**	**<0.001**
**SCD, cm**	1.244	0.673-2.301			
**CAP, dB/m**	1.001	0.995-1.007			
**ATT, dB/cm/MHz**	0.974	0.587-1.615			
