# Supplementary material for: Compensated Advanced Chronic Liver Disease and Steatosis in Patients with Type 2 Diabetes as Assessed through Shear Wave Measurements and Attenuation Measurements
Source: Biomedicines. 2024 Jan 30;12(2):323. doi: 10.3390/biomedicines12020323 (PMC10886655; doi:10.3390/biomedicines12020323)
Supplement: Supplementary file 1 [file biomedicines-12-00323-s001.zip › Supplementary Table S2.pdf]

Supplementary Table S2. Univariate and multivariate logistic regression model assessing independent predictors associated with the SWM>11 kPa (highly suggestive of cACLD). that indicate the presence of cACLD. Significant p-values are bold. **Abbreviations** ALT: alanine aminotransferase; AST: aspartate aminotransferase; ALP: Alkaline phosphatase; APRI: AST to Platelet Ratio Index; ATT: Attenuation coefficient measurement**;** CAP: continuous attenuation parameter; cACLD: compensated advanced chronic liver disease; CRP: C-reactive protein; dB/cm/MHz: decibel per centimeter per megahertz; dB/m: decibels per meter; FIB-4: Fibrosis-4; GGT: gamma glutamyl transferase; g/L: grams per liter; HbA1c: glycated haemoglobin; HDL: high-density lipoprotein; IQR: interquartile range; kPa: kilopascal; LDL: low-density lipoprotein; M: median; µmol/L: micromoles per liter; mmol/L: millimoles per liter; N: number; MASLD: Metabolic dysfunction-associated steatotic liver disease; PT: prothrombin time; SCD: skin to capsule distance; SWM: shear wave measurement; U/L: units per liter; VCTE: vibration-controlled transient elastography; Vs: shear wave speed.

**Dependent variable: SWM> 11 kPa**	**Odds ratio (univariate)**	**95% CI**	**Odds ratio (multivariate)**	**95% CI**	**P value for multivariate analysis**
**Independent variables:**					
**Age, years**	1.019	0.976-1.064			
**Male sex**	0.817	0.344-1.942			
**BMI, kg/m ^{2}**	1.009	0.929-1.096			
**Obesity (BMI>30 kg/m2)**	1.329	0.532-3.317			
**Arterial hypertension**	1.931	0.547-6.816			
**Hyperlipidemia**	0.898	0.334-0.415			
**Smoking**	1.951	0.774-4.921			
**Hematocrit**	1.819	0.0002-13476.6			
**Red cell count, G/L**	0.378	0.136-1.051			
**Platelets, G/L**	**0.977**	**0.967-0.988**	0.965	0.916-1.017	0.18
**PT (%)**	0.98	0.953-1.008			
**Glucose, mmol/L**	1.05	0.908-1.214			
**HbA1c (%)**	0.834	0.559-1.242			
**Creatinine, µmol/L**	**0.971**	**0.946-0.996**	0.971	0.916-1.029	0.33
**AST, U/L**	**1.066**	**1.037-1.095**	1.305	0.933-1.827	0.12
**ALT, U/L**	**1.013**	**1.001-1.025**	0.989	0.941-1.041	0.69

**GGT, U/L**	**1.002**	**1.0-1.004**	0.998	0.993-1.002	0.32
**ALP, U/L**	1.003	0.994-1.012			
**Total cholesterol, mmol/L**	1.239	0.892-1.723			
**Triglycerides, mmol/L**	0.568	0.302-1.067			
**HDL, mmol/L**	**4.188**	**1.072-16.36**	1.254	0.054-29.007	0.89
**LDL, mmol/L**	1.392	0.885-2.191			
**Albumins, g/L**	1.078	0.985-1.179			
**CRP, mg/L, mmol/L**	1.013	0.935-1.098			
**NAFLD fibrosis score, points**	0.015	0.0002-1.103			
**FIB-4, points**	**5.953**	**2.923-12.122**	1.283	0.109-15.143	0.84
**APRI, points**	**409.582**	**41.18-4073.67**	0.0001	0.000001-982.878	0.16
**Fibroscan XL probe**	0.948	0.37-2.425			
**LSM by VCTE, kPa**	**1.542**	**1.318-1.805**	**1.371**	**1.151-1.633**	**<0.001**
**SCD, cm**	1.502	0.73-3.088			
**CAP, dB/m**	1.0009	0.993-1.008			
**ATT, dB/cm/MHz**	0.955	0.483-1.889			
