# Supplementary material for: Compensated Advanced Chronic Liver Disease and Steatosis in Patients with Type 2 Diabetes as Assessed through Shear Wave Measurements and Attenuation Measurements
Source: Biomedicines. 2024 Jan 30;12(2):323. doi: 10.3390/biomedicines12020323 (PMC10886655; doi:10.3390/biomedicines12020323)
Supplement: Supplementary file 1 [file biomedicines-12-00323-s001.zip › Supplementary Table S3.pdf]

Supplementary Table S3. Univariate and multivariate logistic regression model assessing independent predictors associated with the ATT>0.63 dB/cm/MHz (indicative of the presence of liver steatosis). Significant p-values are bold. **Abbreviations** ALT: alanine aminotransferase; AST: aspartate aminotransferase; ALP: Alkaline phosphatase; APRI: AST to Platelet Ratio Index; ATT: Attenuation coefficient measurement**;** CAP: continuous attenuation parameter; CRP: C-reactive protein; dB/cm/MHz: decibel per centimeter per megahertz; dB/m: decibels per meter; FIB-4: Fibrosis-4; GGT: gamma glutamyl transferase; g/L: grams per liter; HbA1c: glycated hemoglobin; HDL: high-density lipoprotein; IQR: interquartile range; kPa: kilopascal; LDL: low-density lipoprotein; M: median; µmol/L: micromoles per liter; mmol/L: millimoles per liter; N: number; MASLD: Metabolic dysfunction-associated steatotic liver disease; PT: prothrombin time; SCD: skin to capsule distance; SWM: shear wave measurement; U/L: units per liter; VCTE: vibration-controlled transient elastography; Vs: shear wave speed.

**Dependent variable: ATT> 0.63 dB/cm/MHz**	**Odds ratio (univariate)**	**95% CI**	**Odds ratio (multivariate)**	**95% CI**	**P value for multivariate analysis**
**Independent variables:**					
**Age, years**	0.981	0.956-1.001			
**Male sex**	1.147	0.669-1.971			
**BMI, kg/m ^{2}**	**1.095**	**1.037-1.157**	0.967	0.852-1.099	0.61
**Obesity (BMI>30 kg/m2)**	**2.901**	**1.630-5.173**	2.287	0.735-7.117	0.15
**Arterial hypertension**	0.533	0.274-1.037			
**Hyperlipidemia**	0.909	0.482-1.716			
**Smoking**	0.945	0.496-1.799			
**Hematocrit**	3.119	0.008-1266.377			
**Red cell count, G/L**	1.224	0.619-2.424			
**Platelets, G/L**	1.003	0.999-1.008			
**PT (%)**	0.995	0.971-1.02			
**Glucose, mmol/L**	1.002	0.909-1.104			
**HbA1c (%)**	1.046	0.869-1.258			
**Creatinine, µmol/L**	0.99	0.979-1.001			
**AST, U/L**	1.005	0.993-1.017			
**ALT, U/L**	1.002	0.996-1.007			

**GGT, U/L**	**1.011**	**1.003-1.018**	1.007	0.999-1.015	0.06
**ALP, U/L**	1.005	0.997-1.014			
**Total cholesterol, mmol/L**	**1.351**	**1.08-1.69**	0.739	0.288-1.897	0.53
**Triglycerides, mmol/L**	**1.51**	**1.334-2.01**	1.167	0.754-1.806	0.49
**HDL, mmol/L**	0.739	0.308-1.771			
**LDL, mmol/L**	**1.338**	**1.009-1.774**	1.628	0.562-4.713	0.37
**Albumins, g/L**	1.002	0.935-1.074			
**CRP, mg/L, mmol/L**	1.002	0.94-1.068			
**NAFLD fibrosis score, points**	1.428	0.184-11.09			
**FIB-4, points**	0.899	0.672-1.204			
**APRI, points**	0.982	0.444-2.17			
**Fibroscan XL probe**	**2.168**	**1.204-3.903**	0.799	0.24-2.662	0.71
**LSM by VCTE, kPa**	**1.046**	**1.0002-1.093**	**1.105**	**1.011-1.208**	**0.03**
**SCD, cm**	**2.186**	**1.298-3.68**	1.112	0.409-3.024	0.83
**CAP, dB/m**	**1.015**	**1.01-1.021**	**1.112**	**1.0002-1.018**	**0.04**
**SWM, kPa**	1.034	0.983-1.087			
